# Impact of Age on Short- and Long-Term Outcomes after Pancreatoduodenectomy for Periampullary Neoplasms

**DOI:** 10.1155/2020/1793051

**Published:** 2020-04-16

**Authors:** Mario Gruppo, Francesca Tolin, Boris Franzato, Pierluigi Pilati, Ylenia Camilla Spolverato, Francesca Zingales, Imerio Angriman, Romeo Bardini

**Affiliations:** ^1^Unit of Surgical Oncology of the Esophagus and Digestive Tract, Veneto Institute of Oncology (IOV-IRCCS), Italy; ^2^Department of Surgery, Oncology and Gastroenterology, University Hospital of Padua, Italy

## Abstract

**Background:**

Although mortality and morbidity of pancreatoduodenectomy (PD) have improved significantly over the past years, the impact of age for patients undergoing PD is still debated. This study is aimed at analyzing short- and long-term outcomes of PD in elderly patients.

**Methods:**

124 consecutive patients who have undergone PD for pancreas neoplasms in our center between 2012 and 2017 were analyzed. Patients were divided into two groups: group I (<75 years) and group II (≥75 years). Demographic features and intraoperative and clinical-pathological data were collected. Primary endpoints were perioperative morbidity and mortality; complications were classified according to the Clavien-Dindo Score. Secondary endpoints included feasibility of adjuvant treatment and overall survival rates.

**Results:**

A total of 106 patients were included in this study. There were 73 (68.9%) patients in group I and 33 (31.1%) in group II. Perioperative deceases were 4 (3.6%), and postoperative pancreatic fistulas were 34 (32.1%). Significant difference between two groups was demonstrated for the ASA Score (*p* = 0.004), Karnofsky Score (*p* = 0.025), preoperative jaundice (*p* = 0.004), and pulmonary complications (*p* = 0.034). No significance was shown for diabetes, radicality of resection, stage of disease, operative time, length of stay, postoperative complications according to the Clavien-Dindo Score, postoperative mortality, pancreatic fistula, and reoperation rates. 69.9% of the patients in group I underwent adjuvant treatment vs. 39.4% of the older ones (*p* = 0.012). Mean overall survival was 28.5 months in group I vs. 22 months in group II (*p* = 0.909).

**Conclusion:**

PD can be performed safely in elderly patients. Advanced age should not be an absolute contraindication for PD, even if greater frailty should be considered. The outcome of elderly patients who have undergone PD is similar to that of younger patients, even though adjuvant treatment administration is significantly lower, demonstrating that surgery remains the main therapeutic option.

## 1. Introduction

Aging is a natural process, and the number of elderly is rapidly growing in western countries. It has been estimated that American elderly are the fastest growing age group and that they will become more than a fifth of the whole population by 2030 [1]. In the United States, pancreatic cancer is the fourth leading cause of cancer death [2–4]. Age-specific incidence rates increase from around the age of 50 years, with the highest incidence in the over 85-year-old age group [5]. The same is true for other periampullary neoplasms, including distal bile duct cancer and ampullary and duodenal cancers [5].

Pancreatoduodenectomy (PD) is still burdened by high rates of morbidity and mortality, ranging from 35% to 51% and from 1% to 6%, respectively [6–8], and the role of age in surgical outcome is still debated [9, 10]. Furthermore, elderly are less likely to receive proper adjuvant chemotherapy, and this could make surgery less effective [11].

The aim of this study was to analyze short- and long-term outcomes of PD on a population of elderly (≥75 years of age) compared with a cohort of younger patients, to determine potential differences on postoperative and oncological outcomes.

## 2. Patients and Methods

124 consecutive patients who have undergone PD for periampullary neoplasms in our center between 2012 and 2017 were analyzed. 18 subjects were excluded because of lack of data or other concomitant procedures. No associated vascular resections were performed. A Whipple resection with pancreatojejunostomy and modified Child's reconstruction was performed in all 106 remaining cases. Pancreas texture was defined as soft or hard depending on manual palpation. At the end of the procedure, 3 tubular drainages were placed close to pancreatic and biliary anastomoses and 1 tubular drainage was placed in the jejunal loop anastomosed to pancreatic remnant and bile duct in order to drain pancreatic juice, bile, and jejunal fluid, according to the institutional reconstruction technique [12]. Patients were divided into two groups: group I (<75 years) and group II (≥75 years). Demographic features and intraoperative and clinical-pathological data were collected; data about octogenarians were analyzed both within the elderly group and separately. TNM staging was reported according to the American Joint Committee on Cancer (AJCC) 7th edition [13]. Primary endpoints were perioperative morbidity and 90-day mortality; complications were classified according to the Clavien-Dindo Score, risk of pancreatic fistula was calculated according to Callery et al.'s validated Fistula Risk Score (FRS) [14], postoperative pancreatic fistula was defined according to International Study Group of Pancreatic Surgery (ISGPS) definitions, and clinically relevant postoperative pancreatic fistula CR-POPF (grade B or C) has been considered as a complication [15]. Secondary endpoints included feasibility of adjuvant treatment and overall survival (OS) rates.

## 3. Statistical Analysis

Continuous data were expressed as median and range, or mean ± standard deviation when appropriate. Rates were expressed as numbers and percentage. In univariate analysis, continuous variables have been compared with a nonpaired *t*-test. Categorical variables have been compared with a *χ*^2^ test. Overall survival (OS) rates were calculated using the method of Kaplan-Meier. Statistical significance was defined as *p* < 0.05. All statistical analyses were performed with IBM SPSS Statistics 24 (IBM, Chicago, IL, United States).

## 4. Results

There were 73 (68.9%) patients in group I and 33 (31.1%) in group II; there were 9 octogenarians (12.3%). 44 PD were performed for pancreatic adenocarcinoma, and 62 patients underwent PD for other periampullary neoplasms (12 duodenal adenocarcinomas, 18 ampullary adenocarcinomas, 9 biliary carcinomas, 8 pancreatic metastases from renal cell cancer, 5 G2-G3 pNETs, 8 IPMN-carcinomas, and 2 solid pseudopapillary neoplasms). Perioperative deceases were 4 (3.6%), including 1 (11.1%) from among the octogenarians, and postoperative pancreatic fistulas were 34 (32.1%) of which 3 (33.3%) were in patients ≥80 years old. Mean overall follow-up was 18.1 months (range 5-56 months); mean follow-up in group I was 19.1 months (range 5-56) and 15.6 months (range 5-36) in group II (*p* = 0.106). Demographic and preoperative clinical data are reported in Table 1; older patients were more prone to be ASA 3 (66.7% vs. 31.5% of younger patients; *p* = 0.004), presented a poorer performace status (mean Karnofsky Score 90% vs. 94.7% in group II; *p* = 0.025), and had higher bilirubin levels (117.66 *μ*mol/L vs. 75.26; *p* = 0.004). Data about octogenarians were comparable to other group II cases.

ASA: American Society of Anesthesiologists; BMI: body mass index.

Intraoperative and pathological data are reported in Table 2: no significant differences were reported for operative time, estimated blood loss, radicality of resection, pancreas consistency, Wirsung dilation, TNM staging, and Fistula Risk Score. Data about octogenarians were comparable to other group II cases.

Postoperative outcome is reported in Table 3; a significant difference was reported only for pulmonary complications (0 in group I vs. 6.1% in older patients, *p* = 0.034; no pulmonary complications occurred in octogenarians). No differences were reported for length of hospital stay, 90-day mortality, and other postoperative complications (cardiovascular diseases, hemorrhages, abdominal collections, surgical site infections, bile leaks, and sepsis). Rates of complications with grade ≥ 3b, according to the Clavien-Dindo Score, were comparable between the groups (8.2% vs. 12.1%; *p* = 0.525), as well as incidence of CR-POPF (30.1% in group I vs. 36.3% in group II; *p* = 0.525). Among octogenarians, complications with the Clavien − Dindo Score ≥ 3b occurred in 1 patient (11.1%), while CR-POPF was detected in 3 cases (33.3%).

POD: postoperative day.

Adjuvant chemotherapy was administered to 69.9% of patients in group I vs. 39.4% of older ones (*p* = 0.012); 22.2% of octogenarians underwent adjuvant treatment.

**Figure 1:**
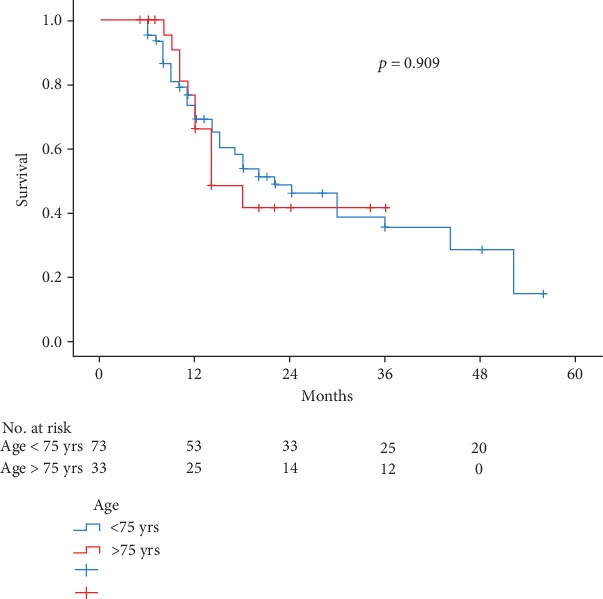
Overall survival of 106 patients who have undergone pancreatoduodenectomy for periampullary neoplasms, according to age stratification.

Overall survival (OS), considering all patients, is reported in Figure 1 and demonstrated no significant differences between the groups with comparable 2-year survival (45.4% in group I vs. 41.2% in group II) and an estimated mean survival of 28.5 months in group I vs. 22.1 in group II; regarding pancreatic ductal adenocarcinoma (PDAC), estimated mean OS in group 1 was 16.8 months (2-year survival 28.3%) vs. 19.1 months (2-year survival 30.6%) in group II (*p* = 0.537).

## 5. Discussion

The elderly population is increasing worldwide. In parallel, the rate of chronic and neoplastic diseases is rising steeply. In developed countries, the accepted definition of elderly population is of subjects ≥ 65 years of age with stratification in three categories: young older (65-74 yrs), older (75-84 yrs), and big older (≥85 yrs). Even more elderly patients suffer from neoplastic diseases and need to undergo major surgical operations. Pancreatoduodenectomy (PD), actually, represents the best curative treatment for periampullary neoplasms, but it is commonly burdened by high rates of morbidity and mortality that make some authors consider this kind of surgery as high risk in elderly people and patients should be carefully selected, even in high-volume centers [10, 16–18]. Furthermore, data on chemotherapy administration in elderly people are lacking [11], and sometimes, the best treatment choice is not easy.

In this study, the “frailty” of elderly patients is confirmed by a significantly higher rate of ASA Score 3 and a poorer performance status compared to younger patients (Table 1); however, intraoperative data did not show significant differences in terms of operative time, blood loss, lymph node retrieval, and radicality of resection, demonstrating that surgical technique and extent of resection is not affected by age of patients [9].

Considering postoperative mortality (90-day mortality), our data suggest a trend towards a worse outcome in the elderly group even though this is without statistical significance; a lack of significance could be related to sample size, but it is important to consider that in-hospital deceases, directly related to surgical operation, were similar (1 in group I and 1 in group II), while the other 2 deaths among older patients occurred from heart failure after discharge.

In the same way, overall postoperative complications were similar between the groups; in particular, no differences were reported for CR-POPF, surgical site infection, postoperative hemorrhage (PPH), abdominal collections, bile leaks, and reoperation rates. A trend towards a higher rate of cardiovascular complications (5.5% vs. 12.1%, *p* = 0.252) and a significantly higher rate of pulmonary complications was reported in the elderly groups (*p* = 0.034); however, these findings do not seem to affect postoperative outcome, as no differences were detected either for length of stay or rates of Clavien − Dindo Score ≥ 3b complications. Our data do not confirm previous reported data of worse outcomes in the elderly population [6, 7, 10, 18–22].

This evidence let us speculate that, probably, reduced functional reserves represent a potential risk factor for cardiovascular and pulmonary complications and, consequently, for mortality [23, 24]. However, a dedicated perioperative management and a perioperative fluid intervention with an individualized “goal-directed” fluid balance could prevent the onset of such disorders in this subset of patients, demonstrating that major surgery, such as pancreatic resections, need good expertise and high-volume series both on the “surgical side” and on anesthesiologic management [23, 25–27].

Data regarding oncological outcomes after pancreatic surgery for malignancies in the elderly are controversial so far. Some authors report that the elderly population shows a worse outcome [5, 17]; however, other groups did not find any significant survival difference [4, 9, 28]. This heterogeneity of results is probably due to selection biases, but our sample also shows a comparable overall survival between older and younger groups. In our opinion, these data are more interesting if related to chemotherapy administration, demonstrating that intention-to-treat surgery still remains the best therapeutic option, even if it is important to consider that adjuvant chemotherapy administration in our sample was gemcitabine-based monotherapy and does not take into account new regimens such as FOLFIRINOX and Gemcitabine-Nabpaclitaxel that could affect outcome significantly [29]. Data regarding access of elderly people to adjuvant chemotherapy are still lacking, but it is commonly accepted that admission to adjuvant treatments is not frequent for elderly patients due to clinical and social aspects, even if they obtain the same surgical oncological benefits [30]. Elderly patients commonly present preexistent comorbidities that reduce functional reserves, worse postdischarge home care and rehabilitation services, and, often, live alone, so the adherence to medical management could be insufficient; all these factors could significantly affect outcome and opportunity to access surgical and medical treatments.

## 6. Conclusions

This study has biases that must be considered, such as the retrospective nature, the size of sample, and the lack of data about patients who did not receive surgery due to comorbidities that probably led us to select the most fit elderly patients for surgery. On the contrary, the strength of this research is a good case matching that can give weight to advanced age as an independent variable. According to our data, advanced age is not an absolute contraindication to PD. Elderly patients fit for surgery can be submitted to PD safely with similar outcome to younger patients in terms of perioperative complications and overall survival, demonstrating that a good case volume, both for surgery and anesthesiology, is determinant for a good perioperative management and outcome. Furthermore, patient selection and recruitment still remain crucial for the therapeutic pathway, above all in order to access and adhere to postoperative medical treatments, claiming the need for more evidence in chemotherapy administration in elderly patients.

## Figures and Tables

**Table 1:** Demographic and preoperative clinical data of 106 patients.

Parameter	Group I (<75 yrs) (73 pts)	Group II (≥75 yrs) (33 pts)	*p*
Age, median (range)	61 (27-72)	78 (75-86)	
Sex, *n* (%)			
Male	39 (53.4)	18 (54.5)	0.915
Female	34 (46.6)	15 (45.5)	
Diabetes, *n* (%)			
Yes	22 (30.1)	9 (27.3)	0.842
No	51 (69.9)	24 (72.7)	
ASA Score, *n* (%)			
1-2	50 (68.5)	11 (33.3)	0.004
3	23 (31.5)	22 (66.7)	
Karnofsky Score, mean (%)	94.7	90	0.025
BMI, mean	22.08 ± 3.77	23.27 ± 4.11	0.225
Preoperative total bilirubin, mean (*μ*mol/L)	75.26 ± 98.11	117.66 ± 147.94	0.004
Preoperative Ca 19-9, mean (kU/L)	7908 ± 46941	11868 ± 53783	0.567
Preoperative serum albumin, mean (g/L)	37.93 ± 6.70	34.73 ± 6.89	0.582

**Table 2:** Intraoperative and pathological data of 106 patients.

Parameter	Group I (73 pts < 75 yrs)	Group II (33 pts ≥ 75 yrs)	*p*
Pathology, *n* (%)			
Pancreatic ductal adenocarcinoma	27 (37)	17 (51.5)	0.160
Others	46 (63)	16 (48.5)	
Pancreatic tissue, *n* (%)			
Hard	24 (32.9)	16 (48.5)	0.125
Soft	49 (67.1)	17 (51.5)	
Wirsung dilation, *n* (%)			
Yes	33 (43.7)	11 (33.3)	0.282
No	40 (56.3)	22 (66.7)	
Radicality, *n* (%)			
R0	57 (78.1)	23 (69.7)	0.325
R1	16 (21.9)	10 (30.3)	
T stage, *n* (%)			
T1-2	59 (80.8)	28 (84.8)	0.238
T3	14 (19.2)	5 (15.2)	
Lymph-nodal status, *n* (%)			
N0	35 (47.9)	14 (42.4)	0.899
N+	38 (52.1)	19 (57.6)	
Operative time (min)	309 ± 9	301 ± 76	0.694
Fistula Risk Score, mean (range)	4.2 (0-8)	3.9 (0-8)	0.439

**Table 3:** Postoperative outcome of 106 patients who have undergone pancreatoduodenectomy for periampullary neoplasms.

Parameter	Group I (73 pts < 75 yrs)	Group II (33 pts ≥ 75 yrs)	*p*
Hospital stay (days)	21 ± 10	23 ± 13	0.452
Reoperation, *n* (%)	5 (6.8)	1 (3.1)	0.659
90-d deaths, *n* (%)	1 (1.4)	3 (9.1)	0.088
Pulmonary complications, *n* (%)	0 (0)	2 (6.1)	0.034
Cardiovascular complications, *n* (%)	4 (5.5)	4 (12.1)	0.252
Other complications, *n* (%)	22 (30.1)	9 (27,3)	0.764
Clavien − Dindo Score ≥ 3B, *n* (%)	6 (8.2)	4 (12.1)	0.525
Pancreatic fistula (grades B-C), *n* (%)			
No	51 (69.9)	21 (63.7)	0.525
Yes	22 (30.1)	12 (36.3)	

## Data Availability

The clinical, demographic and pathological data used to support the findings of this study are included within the article.
